# Supplementary material for: Subwavelength terahertz imaging via virtual superlensing in the radiating near field
Source: Nat Commun. 2023 Oct 18;14:6393. doi: 10.1038/s41467-023-41949-5 (PMC10584837; doi:10.1038/s41467-023-41949-5)
Supplement: Supplementary file 1 — Supplementary Information [file 41467_2023_41949_MOESM1_ESM.pdf]

Supplementary Information:
"Subwavelength terahertz imaging via virtual superlensing in the radiating near field"

Alessandro Tuniz and Boris T. Kuhlmey
Institute of Photonics and Optical Science, School of Physics,
University of Sydney, NSW 2006, Australia and
The University of Sydney Nano Institute, The University of Sydney, NSW 2006, Australia[*]

This document provides supplementary material to "Subwavelength terahertz imaging via virtual superlensing in the radiating near field". We provide supplementary notes of the noise limit derivation and non-perturbative imaging, a supplementary table with simulation and experimental parameters, and supplementary figures showing samples and experimental apparatus, distance calibration, the signal-to-noise ratio for each experiment, numerical simulations of the near fields and their reconstruction, measurements and simulations of phase fluctuation effects on terahertz imaging, perturbation of resonant frequency due to an imaging tip, and an example perturbation of a high-Q resonator due to a probing tip in the near field.

SUPPLEMENTARY NOTE 1

Noise limit derivation

Defining $k_\perp^2 = k_x^2 + k_y^2$ so that $k_z = \sqrt{k_0^2 - k_\perp^2}$, let $\tilde{T}$ be the transfer function of the fields along z:

$$\tilde{T}(k_x, k_y, z) = \exp(ik_z z) \tag{1}$$

such that $\tilde{E}(k_x, k_y, z) = \tilde{T}(k_x, k_y, z)\tilde{E}(k_x, k_y, z = 0)$ (which is propagation in free space) and conversely $\tilde{E}(k_x, k_y, z = 0) = \tilde{T}(k_x, k_y, -z)\tilde{E}(k_x, k_y, z)$ (which is the superlensing procedure). For propagating fields $k_\perp < k_0$ and

$$|T(k_\perp < k_0)| = 1. \tag{2}$$

For evanescent fields, $k_\perp > k_0$ and

$$|T(k_\perp > k_0)| = \exp(\pm|k_z|z), \tag{3}$$

with a $-$ sign for the evanescent decay (transfer from object to measurement distance) and $+$ sign for the superlensing procedure.

At a distance z from the source, the spatial Fourier transform of the measured field is given by

$$\tilde{E}_M(k_x, k_y) = \tilde{E}_m(k_x, k_y) + \tilde{\delta}_m(k_x, k_y) \tag{4}$$

where $\tilde{E}_m(k_x, k_y)$ is the actual field (*i.e.*, in the absence of noise), and $\tilde{\delta}_m(k_x, k_y)$ is the noise due to the measurement instrument, where typically $|\tilde{\delta}_m(k_x, k_y)| \ll \max|\tilde{E}_m(k_x, k_y)|$. The reconstructed field is given by

$$\tilde{E}^{\rm SL}(k_x, k_y) = T(k_x, k_y, -z)\tilde{E}_m(k_x, k_y) + \tilde{T}(k_x, k_y, -z)\tilde{\delta}_r(k_x, k_y) \tag{5}$$

The first product is exactly the object field we wish to obtain, while the second term is the noise in the reconstructed field $\tilde{\delta}^{\rm SL} = \tilde{\delta}_m \exp(|k_z z|)$, where we omit the explicit (k_x, k_y) dependence for brevity. Compared to the measurement noise, the (amplitude) noise penalty in dB of the procedure is thus

$$\Delta_{\rm noise} = 10\log_{10}\left|\frac{\tilde{\delta}^{\rm SL}}{\tilde{\delta}_m}\right| = 10\log_{10}(\exp(k_z z)) = \frac{10 k_z z}{\log(10)}. \tag{6}$$

[*] alessandro.tuniz@sydney.edu.au

When the noise penalty exceeds the initial signal to noise ratio of the measurement SNR, the image becomes dominated by noise. This occurs when

$$\mathrm{SNR} < \Delta_{\mathrm{noise}} = \frac{10z\sqrt{k_\perp^2 - k_0^2}}{\log(10)} \tag{7}$$

The right hand term increases steadily with $k_\perp$, and the limiting value of $k_\perp = k_{\max}$ at which equality is achieved can be obtained by rearranging Eq. (7) as

$$k_{\max} = k_0\sqrt{1 + \left(\frac{\lambda}{z}\frac{\log 10}{20\pi}\mathrm{SNR}\right)^2}, \tag{8}$$

which corresponds to Eq. (4) in the main manuscript. Filtering out spatial frequencies higher than $k_{\max}$ ensures that the noise penalty due to the superlensing procedure does not generate noise levels above the measurement's signal level, and thus ensures clear images.

SUPPLEMENTARY NOTE 2

Non-perturbative imaging

Even without applying our virtual superlens correction to the decay of evanescent fields, assuming one has appropriate experimental equipment such as a THz oscillating s-SNOM tips, spatial resolution can be improved almost indefinitely by bringing the imaging probe as close as possible to the objects to be imaged. However, any imaging device or scanning tip is a scatterer and thus affect the fields that are to be imaged. An example where this can be particularly problematic is that of high-Q cavities: Bringing in the scattering tip too close will add a loss pathway and thus reduce the Q value and also shift the resonance frequency. This makes it difficult to image high-Q modal fields precisely without affecting the resonance. The virtual superlens approach makes it possible to increase resolution with a considerable reduction in perturbation of the fields. A simple perturbative approach shows that the relative shift in resonance frequency $\Delta f_0/f_0$ due to perturbation by an imaging probe can be reduced by orders of magnitude when using the superlens method compared to direct imaging:

Consider a mode of a resonator with high quality factor Q, with modal fields locally having characteristic transverse wavenumber $k_{\perp\mathrm{mode}}$ along the surface of the resonator, that is the peak of the energy distribution has spatial frequency component $k_{\perp\mathrm{mode}}$. However, the modal fields also have higher spatial frequency details (for example due to holes in a photonic crystal, or due to the presence of a smaller plasmonic resonator) that one wishes to image. To do so, a probe is introduced in the near field, at distance z. The field locally decays as $E \propto e^{-|k_z|z}$ with $k_z = \sqrt{k_{\perp\mathrm{mode}}^2 - k_0^2}$. We model the probe as having a real polarizability α that is proportional to $(\varepsilon_r - 1)\varepsilon_0 V$, where V is the interaction volume of the probe and ε_r its relative permittivity – the exact expression of α depends on details of the probe geometry and its orientation in the field, and would become complex if absorption and re-radiation by the dipole are taken into account. First order perturbation theory leads to a relative shift of resonant frequency given by the ratio of energy in the induced dipole of the tip $\alpha|E(z)|^2$ divided by the total energy in the (unperturbed) cavity U_0[1]:

$$\frac{\Delta f_0}{f_0} \simeq -\frac{\alpha|E(z)|^2}{U_0}. \tag{9}$$

The relative frequency shift thus scales as $|E(z)|^2$, that is as $|E(0)|^2 e^{-2z|k_z|}$.

Without amplification of evanescent waves, imaging a spatial frequency $k_\perp$ requires the tip to be almost in contact with the cavity so that $|k_z|z \ll 1$, and the frequency shift is then

$$\frac{\Delta f_{\text{no amplification}}}{f_0} \simeq -\frac{\alpha|E(0)|^2}{U_0}. \tag{10}$$

From Eq. (7), using the virtual superlensing amplification of evanescent waves, to obtain an image with resolution limited by spatial frequency $k_{\perp\mathrm{image}}$ the probe can be set back to a distance $z = \mathrm{SNR}\frac{\log(10)}{10\sqrt{k_{\perp\mathrm{imaging}}}}$, the shift in resonance is then reduced by

$$\frac{\Delta f_{\text{virtual superlens}}}{\Delta f_{\text{near field tip}}} = \frac{|E(z)|^2}{|E(0)|^2} = e^{-2|k_z|z} = e^{-2\mathrm{SNR}\frac{\log(10)}{10}\frac{k_{z,\mathrm{mode}}}{k_{z,\mathrm{image}}}} \tag{11}$$

The right hand term simplifies to the amplitude signal-to-noise in linear units raised to the power $2k_{z,\mathrm{mode}}/k_{z,\mathrm{imaging}}$. Supplementary Figure 6 shows an example of the reduction in frequency perturbation that can be achieved using a the virtual superlens when imaging fields of a cavity with $k_{\perp,\mathrm{mode}} = 2k_0$, as a function of maximal spatial frequency to be imaged, assuming SNR=20dB. The perturbation of the resonance due to the tip is reduced by several orders of magnitude, demonstrating the virtual superlens technique enables non-perturbative imaging of high-Q cavities.

Supplementary Figure 7 shows an example of the kind of scanning-antenna-induced perturbation that can be avoided using the superlensing procedure: The whispering gallery mode of a silicon high-Q cylindrical resonator is evanescently excited by the mode of a silica slab waveguide. A near field antenna, modelled as the 50 μm substrate slab typical near-field antennas are on (*without* the metallic dipole that would add additional perturbation) is scanned in the x and y directions. The characteristic spatial frequency of the modal fields is of order $k_\perp \simeq 2.4k_0$, which can easily be picked up at a 400 μm distance using the superlens procedure, but would require the near-field antenna to be almost touching to be imaged directly. The simulation show the resonant frequency, resonance width, and field magnitude and phase are all substantially affected by a scanning tip at any distance less than 100 μm, whereas measuring fields at 400 μm leaves fields and resonance unperturbed. Similar effects were recently seen in experimental data of imaging metal nanoresonators using an optical s-SNOM [2].

SUPPLEMENTARY TABLE

Simulation and experimental spatial parameters

Supplementary Table I contains a summary of the step size in the spatial domain Δx and Δy, and the number of points N_x and N_y in x and y respectively, associated with the data shown in Figs. 2–5 of the main manuscript. The window size is given by $X_w = N_x\Delta x$ and $Y_w = N_y\Delta y$. The maximum spatial frequency for each coordinate can then be computed by $K_x^{\max} = \frac{\pi}{\Delta x}$ and $K_y^{\max} = \frac{\pi}{\Delta y}$, and the spatial frequency step size is given by $\Delta K_x = \frac{2\pi}{X_w}$ and $\Delta K_y = \frac{2\pi}{Y_w}$.

Data set	N_x	N_y	Δx [μm]	Δy [μm]
Double slit simulation (Figs. 2–3)	1000	N.A.	10	N.A.
Double aperture experiment (Fig. 4)	81	51	50	50
x-polarized “THZ” letters experiment (Fig. 5)	241	161	25	25
y-polarized “THZ” letters experiment (Fig. 5)	201	161	25	25
x-polarized “THZ” letters simulation (Suppl. Fig. 4)	601	401	10	10
y-polarized “THZ” letters simulation (Suppl. Fig. 4)	601	401	10	10

Supplementary Table I.

SUPPLEMENTARY FIGURES

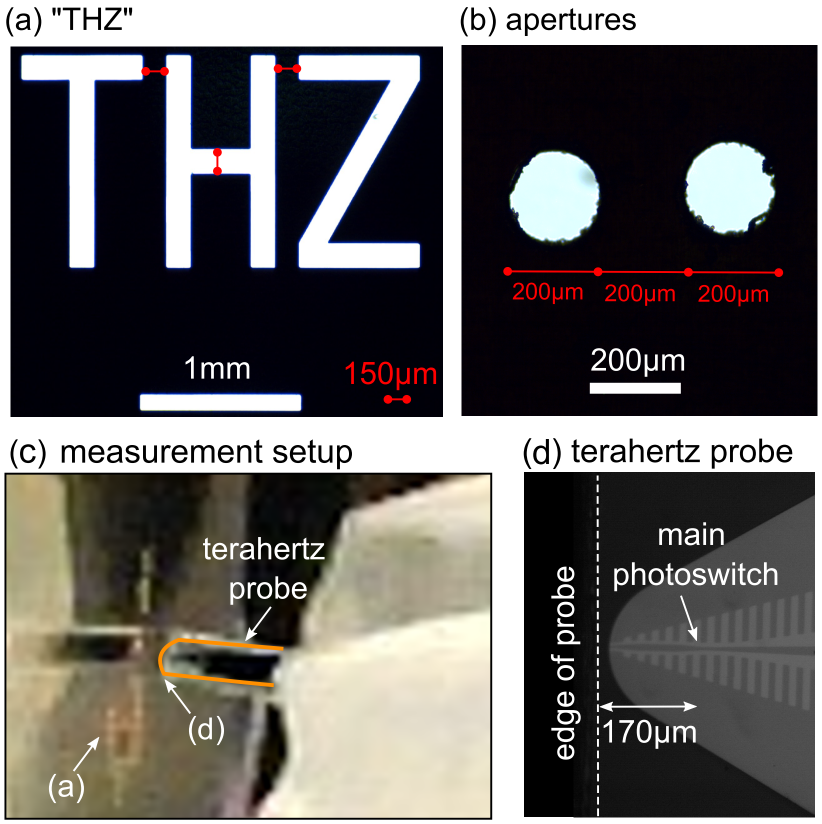

Supplementary Fig. 1. Optical microscope images of (a) the laser-machined "THZ" letters used in Fig. 4 of the manuscript, and (b) the two apertures used for the experiments in Fig. 3 of the manuscript, highlighting the minimum feature sizes in each case. Black: metal; white: air gaps. (c) Photograph of the near-field terahertz probe as it scans the surface of the sample. The terahertz field propagates from the left, is scattered by the sample towards the right, and is measured by the probe. See Ref. [3] for further details of the experimental setup. (d) Microscope image of the near-field terahertz probe, showing the location of its main photoswitch, which is at a nominal distance of 170 μm from the edge of the probe – this also means for this probe used at right angle, only object-to-photoswitch distances $L > 170\mu$m are achievable. Note that small variations in the alignment of the pulses used in the near field detector module – performed anew before starting each scan – can affect both L and the SNR.

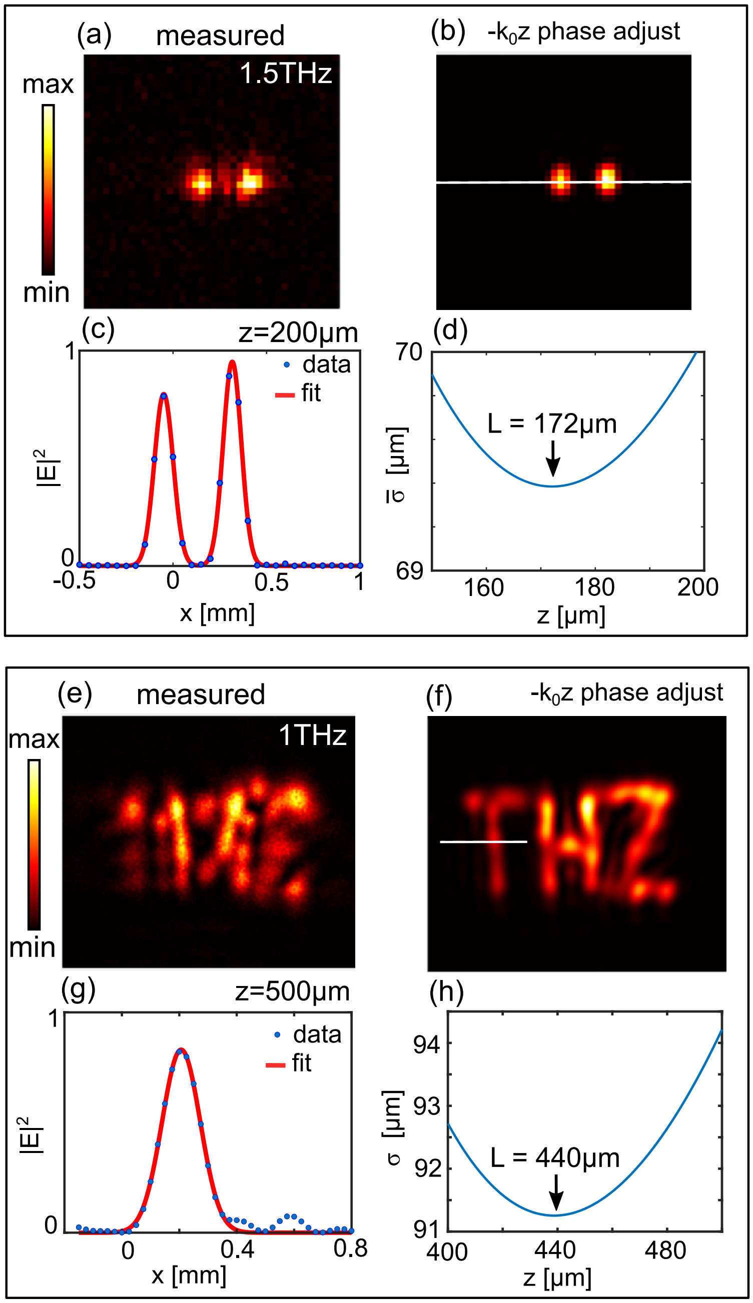

Supplementary Fig. 2. Retrieval of the sample-to-switch distance $z = L$. (a) Measured $|E_x|$ at 1.5 THz for the two-aperture experiment, where the spatial frequencies which resolve the aperture features are propagating. (b) Example image when the phase of $|\tilde{E}_x|$ is adjusted by $-k_0 z$, where $z = 200\,\mu$m. We consider its field magnitude through the apertures' center (white dotted line), and show it as blue circles in (c). Red curves in (c) show a Gaussian fit to a double Gaussian function, used to obtain the average standard deviation $\overline{\sigma}$. (d) Average fitted $\overline{\sigma}$ as a function of z. (e)-(h) Same as (a)-(d), performed for the "THZ" sample. Here we use a single feature, highlighted by the white line in (f), and fit a single gaussian. In this case, $z = L = 440\,\mu$m. In both cases, this numerical processing of our experimental data functionally corresponds to adjusting the focus of a conventional lens, until the sharpest image is obtained.

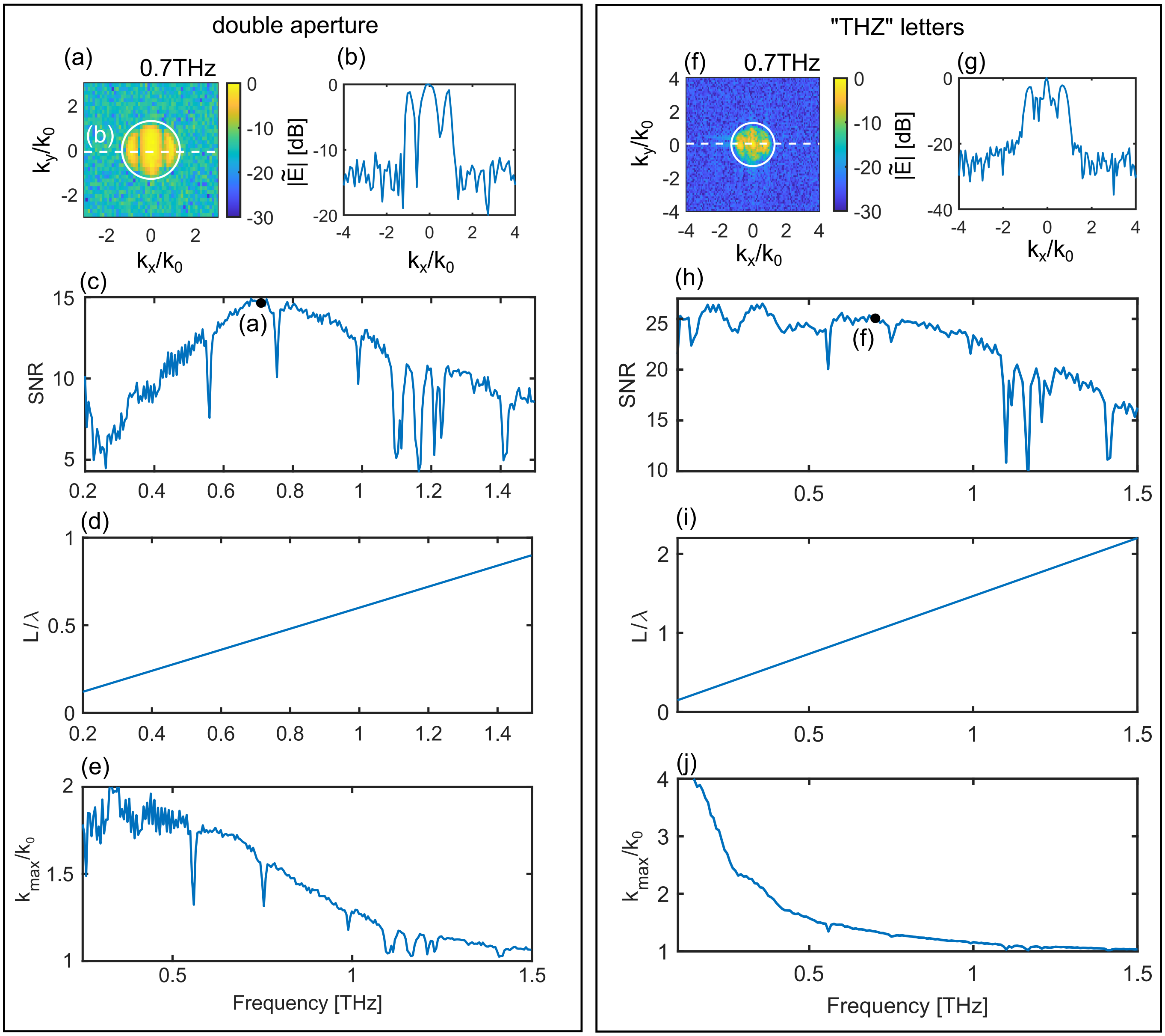

Supplementary Fig. 3. Overview of the method for obtaining the experimental $k_{\max}/k_0$ as a function of frequency for the double aperture experiment of Fig. 4 of the manuscript. (a) Measured $|\tilde{E}_x|$ as a function of k_x/k_0 and k_y/k_0 at an example frequency 0.7 THz. (b) $|\tilde{E}_x|$ as a function of k_x for $k_y = 0$ (white dashed line in (a)), where SNR = 14 dB. (c) Extracted SNR = $\max(|\tilde{E}_x|)/\langle|\tilde{E}_x|(|\mathbf{k}| > k_0)\rangle$ as a function of frequency. The black circle shows the value obtained from (a). The boundary between the propagating and evanescent regions, used in the SNR measurement, is shown as a white circle in (a). Note the maximum SNR of 14 dB at 0.7 THz. (d) Associated L/λ as a function of frequency. (e) Resulting $k_{\max}/k_0$ as a function of frequency, calculated using Eq. (4) of the manuscript. Here $k_{\max}/k_0$ =1–1.8 between 0.2–1.5 THz. (f)–(j) Same as (a)–(e), applied to the "THZ" letters experiment shown in Fig. 5 of the manuscript. Note that, although the sample-to-detector distance L is about twice as long here compared to the double-aperture experiment (implying that high spatial frequencies have decayed more), the SNR reaches values that are nominally 10 dB higher so that higher spatial frequencies can be amplified ($k_{\max}/k_0 > 3$), as per Eq. (4) of the manuscript.

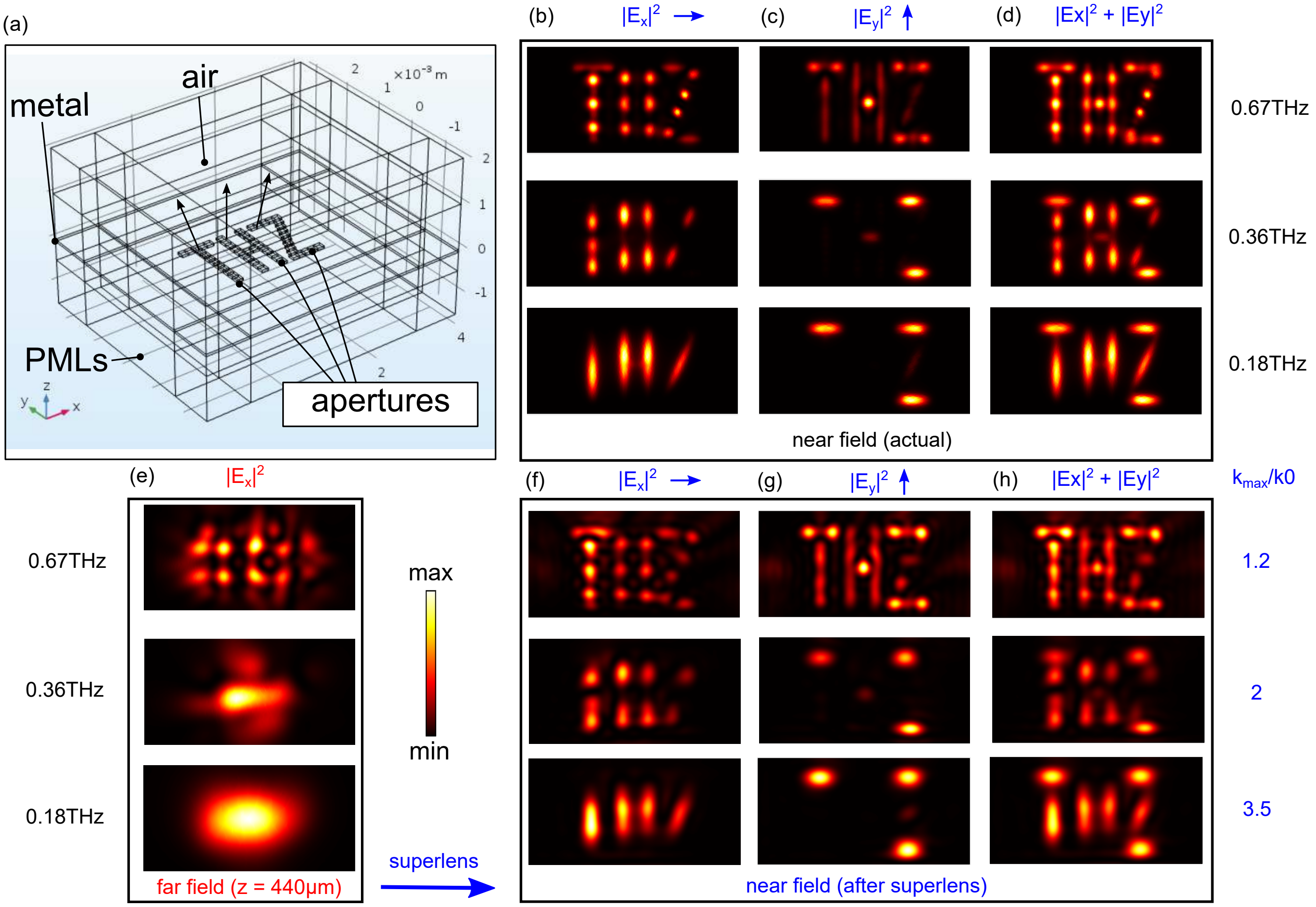

Supplementary Fig. 4. 3D finite element simulations (COMSOL) modelling the field emerging from the letters "THZ" of Fig. 4 in the manuscript, and comparing the superlens procedure result with the near field directly taken from the simulations. (a) Our model replicates the sample used, and considers the electric field emerging from apertures within a metal sheet (metal thickness: $50\,\mu$m; aperture width: $150\,\mu$m) suspended in air. Perfectly matched layers (PMLs) suppress reflections at the boundaries. (b) Simulated $|E_x|^2$, (c) $|E_y|^2$, and (d) their sum $|E_x|^2 + |E_y|^2$ at a distance $z = 50\,\mu$m from the metal sheet. (e) Simulated $|E_x|^2$ at a distance $z = 440\,\mu$m from the metal sheet, for the frequencies as labelled. Also shown are the images obtained when applying the superlens procedure to the numerically calculated fields at $z = 440\,\mu$m, showing the resulting (f) $|E_x|^2$ (d) $|E_y|^2$, and (e) $|E_x|^2 + |E_y|^2$, with k_{max}/k_0 as labelled, for comparison with our experiment. Note that the directly simulated near-fields are comparable to those obtained after applying the superlens procedure in the radiating near field, and display many of the subtle features observed in our experiment, *cf.* Fig. 4 of the manuscript. Each window area is $4\,\text{mm} \times 2\,\text{mm}$.

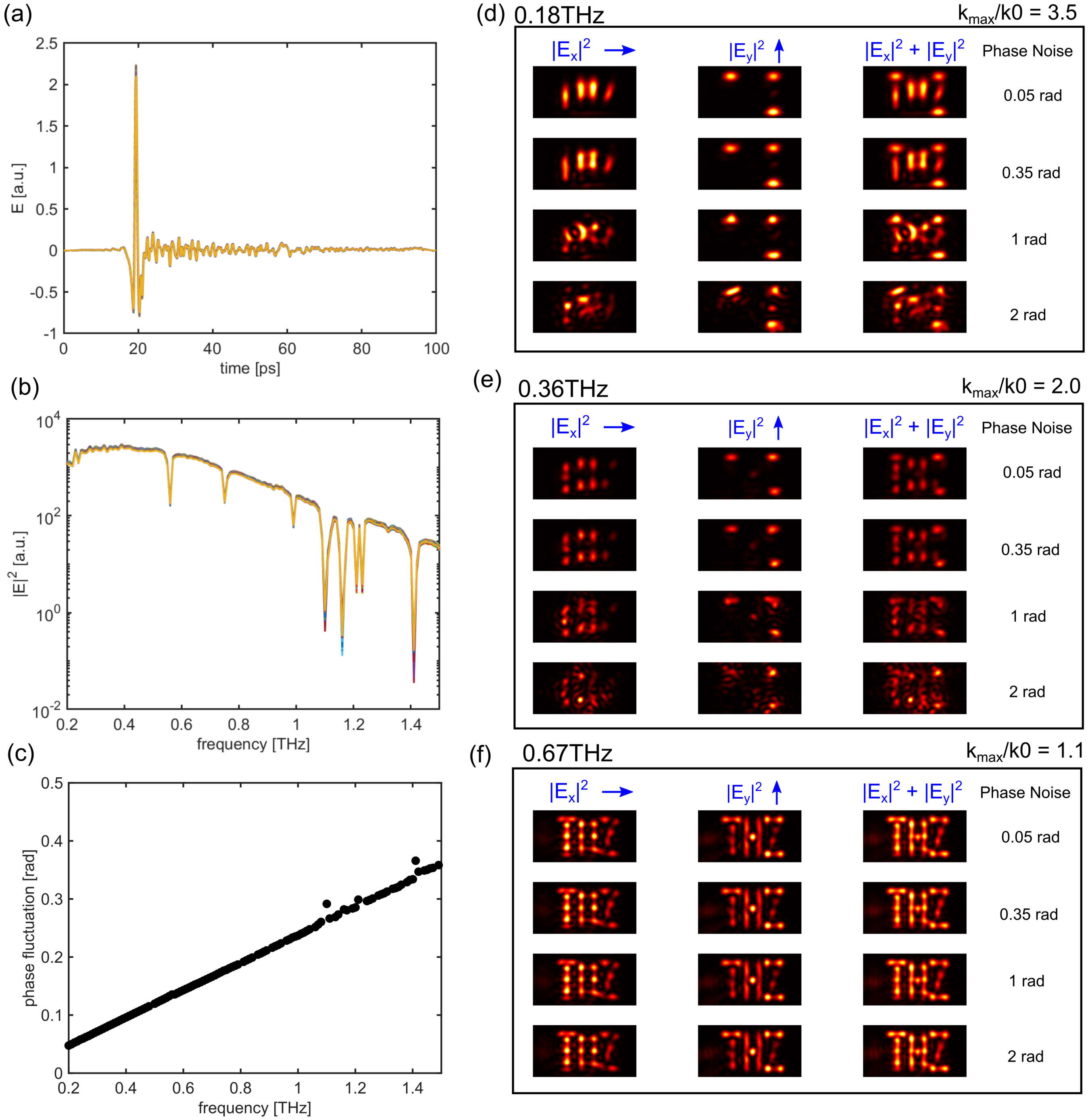

Supplementary Fig. 5. Phase fluctuation effects on terahertz imaging. (a) Example consecutive measurements of the terahertz electric field (850 measurements taken over 70 minutes) (b) Associated intensity for all 850 measurements, obtained from a Fourier Transform of each pulse in (a). (c) Phase fluctuation at each frequency, obtained from the standard deviation of the phase of each data point in (b). Associated effect of phase noise on superlensing for different levels of phase noise at (d) 0.18 THz, (e) 0.36 THz and (f) 0.67 THz, using the simulation data from Supplementary Fig. 4, adding phase noise as labelled, and with $k_{\max}/k_0$ as labelled. Within the measured range of phase noise, the effect of adding phase noise is small. The procedure becomes even more robust to noise for lower values of $k_{\max}/k_0$, where the super lens procedure has smaller amplification factors increasing noise levels.

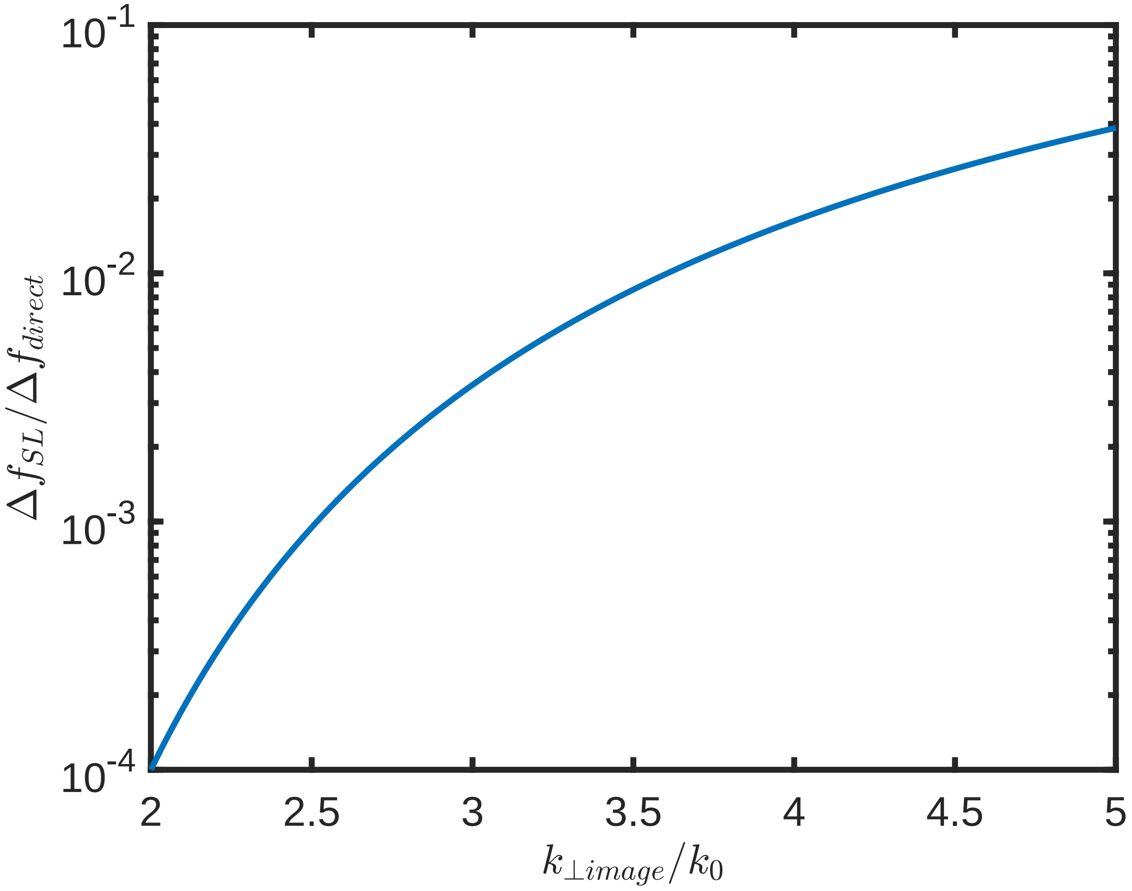

Supplementary Fig. 6. Perturbation of resonant frequency due to an imaging tip: Ratio of frequency perturbation when using the virtual superlens to frequency perturbation with a tip in direct contact without virtual superlens, as a function of desired normalized spatial frequency. Here the cavity mode's dominant spatial frequency is $k_{\perp\mathrm{mode}} = 2k_0$ corresponding to an effective refractive index of 2, and SNR = 20dB.

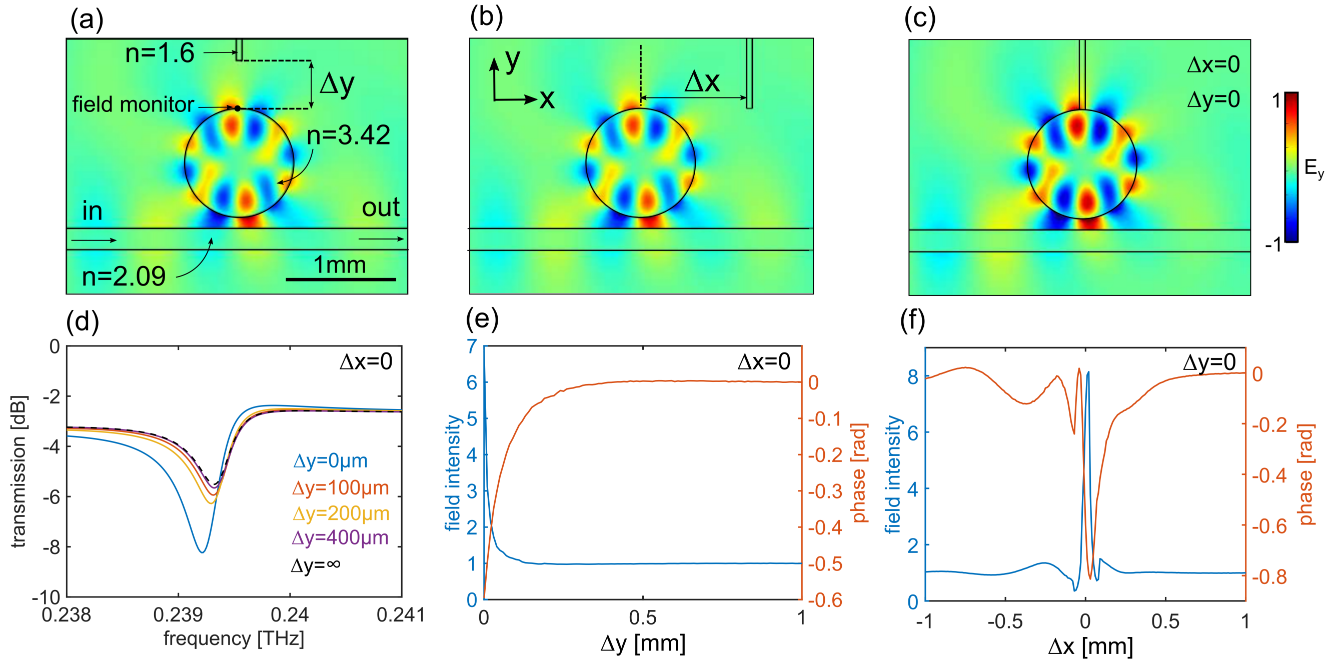

Supplementary Fig. 7. Example of perturbation of a high Q-resonator due to a small probing tip in the near-field. The top row show 2D simulations of the y-component of the electric field for the TM mode propagating through a silica slab waveguide ($n = 2.09$; slab width: $200\,\mu$m) at 0.2932 THz in near-contact with a silicon cylinder ($n = 3.42$; silicon diameter: 1 mm). The near field antenna is modelled as a slab ($n = 1.6$; slab thickness: $50\,\mu$m). A field monitor is placed on the upper surface of the cylinder (black dot). (a) $\Delta x = 0\,\mu$m, $\Delta y = 200\,\mu$m. (b) $\Delta x = 1$ mm, $\Delta y = 0\,\mu$m. (c) $\Delta x = 0\,\mu$m, $\Delta y = 0\,\mu$m. (d) Calculated transmission though the silica waveguide as a function of frequency, in dB, when the antenna is centered in x and moved in y as labelled. Note the shift in resonant frequency and resonance width. (e) and (f) show the normalized field intensity (blue) and phase (red) at 0.2392 THz at the fixed location of the field monitor as the position of the probing antenna is varied along y (e) and along x (f). Note the 7-8 fold increase in field intensity as the antenna is brought in contact with the cylinder, accompanied by phase change of nearly one radian. The field distribution is thus clearly perturbed by the tip when it is scanned at distances that allow to pick up the near-field directly. In contrast, placing the antenna at a vertical distance of $\Delta y = 400\,\mu$m from the resonator, as per our superlens procedure, has a negligible effect on the resonance (d) and field intensity (e), allowing to measure the true field at the unperturbed resonance.

SUPPLEMENTARY REFERENCES

[1] J. A. Kong, *Electromagnetic wave theory* (John Wiley and Sons, 1986).
[2] M. Esmann, S. F. Becker, J. Witt, J. Zhan, A. Chimeh, A. Korte, J. Zhong, R. Vogelgesang, G. Wittstock, and C. Lienau, Vectorial near-field coupling, Nature Nanotechnology **14**, 698 (2019).
[3] A. Stefani, B. T. Kuhlmey, J. Digweed, B. Davies, Z. Ding, H. Zreiqat, M. Mirkhalaf, and A. Tuniz, Flexible terahertz photonic light-cage modules for in-core sensing and high temperature applications, ACS Photonics **9**, 2128 (2022).
